# Development of a Computerized 4-D MRI Phantom for Liver Motion Study

**DOI:** 10.1177/1533034617723753

**Published:** 2017-08-09

**Authors:** Chunhao Wang, Fang-Fang Yin, W. P. Segars, Zheng Chang, Lei Ren

**Affiliations:** 1 Department of Radiation Oncology, Duke University Medical Center, Durham, NC, USA; 2 Medical Physics Graduate Program, Duke Kunshan University, Kunshan, Jiangsu, China; 3 Department of Radiology, Duke University Medical Center, Durham, NC, USA

**Keywords:** MRI, computerized phantom, liver, organ motion, deformable registration

## Abstract

**Purpose:**

To develop a 4-dimensional computerized magnetic resonance imaging phantom with image textures extracted from real patient scans for liver motion studies.

**Methods:**

The proposed phantom was developed based on the current version of 4-dimensional extended cardiac-torso computerized phantom and a clinical magnetic resonance scan. Initially, the extended cardiac-torso phantom was voxelized in abdominal–chest region at the end of exhalation phase. Structures/tissues were classified into 4 categories: (1) Seven key textured organs, including liver, gallbladder, spleen, stomach, heart, kidneys, and pancreas, were mapped from a clinical T1-weighted liver magnetic resonance scan using deformable registration. (2) Large textured soft tissue volumes were simulated via an iterative pattern generation method using the same magnetic resonance scan. (3) Lung and intestine structures were generated by assigning uniform intensity with proper noise modeling. (4) Bony structures were generated by assigning the magnetic resonance values. A spherical hypointensity tumor was inserted into the liver. Other respiratory phases of the 4-dimensional phantom were generated using the backward deformation vector fields exported by the extended cardiac-torso program, except that bony structures were generated separately for each phase. A weighted image filtering process was utilized to improve the overall tissue smoothness at each phase.

**Results:**

Three 4-dimensional series with different motion amplitudes were generated. The developed motion phantom produced good illustrations of abdominal–chest region with anatomical structures in key organs and texture patterns in large soft tissue volumes. In a standard series, the tumor volume was measured as 13.90 ± 0.11 cm^3^ in a respiratory cycle and the tumor’s maximum center-of-mass shift was 2.95 cm/1.84 cm on superior–inferior/anterior–posterior directions. The organ motion during the respiratory cycle was well rendered. The developed motion phantom has the flexibility of motion pattern variation, organ geometry variation, and tumor modeling variation.

**Conclusions:**

A 4-D computerized phantom was developed and could be used to produce image series with synthetic magnetic resonance textures for magnetic resonance imaging research of liver motion.

## Introduction

A computerized phantom has been developed and extensively used as a powerful tool for preliminary demonstration, testing, evaluation, and improvement of novel medical imaging techniques.^1^ Computerized phantoms present a number of distinguished advantages over patient samples, including (1) convenience for implementation, (2) information of human anatomy and physiology for use as “gold standard” for evaluations of the imaging techniques,^2^ and (3) flexibilities in simulating different human anatomical and respiratory motions to study the effects of each individual factor on the accuracy of the imaging techniques. Previously, a 4-dimensional (4-D) digital extended cardiac-torso (XCAT) human phantom was developed for multimodality imaging research.^3^ The XCAT phantom consisted of a series of modeled organs with a high level of human anatomical detail using nonuniform rational B-splines (NURBS) surfaces. Patient respiratory and cardiac motions were modeled in 4-D-XCAT based on the information obtained from patient 4-D tagged magnetic resonance imaging (MRI) and 4-D computed tomography (CT) data. As it was based upon human anatomy and the flexibility of organ modeling, 4-D XCAT was widely used for high-resolution X-ray imaging research (X-ray radiograph and CT) and low-resolution nuclear medicine imaging research (Positron Emission Tomography [PET] and Single-Photon Emission Computed Tomography [SPECT]) in the field of medical imaging studies.^4–8^

Magnetic resonance imaging is a critical imaging modality both for diagnostic and for therapeutic applications mainly due to its superior soft tissue contrast at zero risk of ionizing radiation hazard. In radiation therapy, different 3-dimensional/4-D MRI techniques have been developed recently for motion assessment, on-board target motion tracking, and treatment response assessment.^9–17^ Extended cardiac-torso has been used in some pilot studies as a valuable tool to evaluate the accuracy of the imaging techniques developed. However, the XCAT phantom currently could only simulate uniform signal intensity in each organ for pseudo-MR contrast without soft tissue textures.^18^ The lack of anatomical details and soft tissue patterns in these simple approaches may cause potential problems in the techniques that are image intensity dependent, such as iterative MR reconstruction and k-space acquisition optimization.^19,20^ The simulation results were also less relevant to the clinical situations.

In this work, a 4-D liver computerized phantom was developed based on the up-to-date version of the 4-D XCAT computerized phantom. In addition to the flexibility of motion pattern variation, organ geometry variation, and tumor modeling variation that are inherent in XCAT, the developed phantom has synthesized anatomical details and soft tissue patterns extracted from clinical MR images. It can potentially become a valuable tool for 4-D MRI studies about liver motion.

## Materials and Methods

### Patient Scan and XCAT Phantom

Magnetic resonance textures in the 4-D liver phantom were extracted from real patient MR images. A selected preradiotherapy liver MR scan was anonymized and used in this work. The MR scans were acquired using a 1.5-T Magnetom scanner (Siemens Medical Systems, Erlangen, Germany) with a dedicated phase-array body coil at axial view. A volumetric interpolated breath-hold exam (VIBE) sequence was adopted for T1-weighted imaging.^21^ The key acquisition parameters were TR/TE (Repetition time/Echo time) = 4.45/1.40 milliseconds, flip angle = 15°, in-plane field of view (FOV) = 400 × 400 mm2, slice thickness = 4 mm, number of slice = 72, image matrix = 256^2^ (interpolation, 512^2^), Number of acquisition (NEX) = 1. The VIBE protocol was selected because it is the standard protocol for liver disease assessment in our radiation oncology clinic. Seven organs with unique anatomical details, including the liver, gallbladder, spleen, pancreas, stomach, kidneys, and heart (as shown in Table 1), were contoured in a radiation treatment planning system. A region of interest (ROI) was manually contoured in a larger chunk of muscle for tissue texture random generation in the study.

**Table 1.** List of Defined Structures in the Phantom.^a^

Category I			Category II			Category III		Category IV	
	A	B		A	B		A		A
Liver	95	160	Muscle	40	140	Lungs	18	Rib	32
Gallbladder	82	160	Bodyb	30	80	Airway tree	235	Spine	38
Pancreas	100	112	Static marrowc	30	80	Intestine air	40	Cord	195
Spleen	25	170	Intestine wall	30	85	Esophagus wall	136	Cortical bone	28
Stomachd	85	140				Air	40	Cartilage	140
Kidneyse	90	105						Mobile marrow	135
Heart	45	150							
Pericardium									
Myocardium	65	150							
Heart blood	110	150							

Abbreviations: MR, magnetic resonance; XCAT, extended-cardiac-torso.

^a^A and B are modeled MR signal intensity values with arbitrary units.

^b^Stomach is modeled by stomach wall and stomach content in the XCAT.

^c^Left kidney and right kidney are modeled separately. Each kidney is modeled by the cortex and medulla in the XCAT.

^d^Body is defined as the background tissue not classified as anything else.

^e^In the XCAT, Static marrow and mobile marrow are modeled together as bone marrow. Static marrow is defined as the immobile parts (next to the spine) of the bone marrow. Mobile marrow is defined as moving parts (next to the rib and the cortical bone) of the bone marrow.

**Figure 1.**
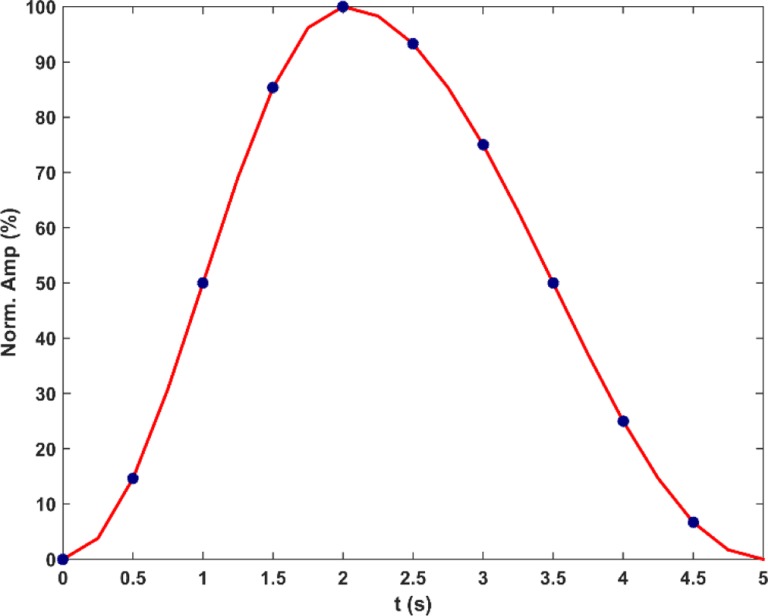
The reference motion–time curve in this phantom. The vertical axis represents the percentage of motion amplitude normalized to the maximum displacement between end of exhalation (EOE, t = 0.0 seconds) and end of inhalation (EOI, t = 2.0 seconds). Blue dots indicate the time points that are corresponding to the generated 10-phase volumes. Both diaphragm motion and anterior–posterior (AP) chest motion are modeled by this curve.

In the XCAT phantom, a region covering the chest and abdomen was defined. In the activity mode, each structure in the defined region was assigned with a unique activity number to label its binary mask for segmentation. The 4-D series of structure masks containing 10 phases in a respiratory cycle (5 seconds) was then generated using the reference motion–time curve, as shown in Figure 1. Each phase volume was composed of 256 × 256 × 150 voxels with an isotropic 1.6719^3^ mm^3^ voxel size. The reference anatomical geometry in this work and other input parameters can be found in Supplementary Material I, which summarized the input file with parameters that are defaults or commonly adopted ones in XCAT-related research. The end of exhalation (EOE) phase volume was the first volume in the 4-D series and was selected as the reference volume. The rest of the phase volumes was considered as deformations of the reference volume.

### Generating Synthetic MRI Volume at the Reference Phase

**Figure 2.**
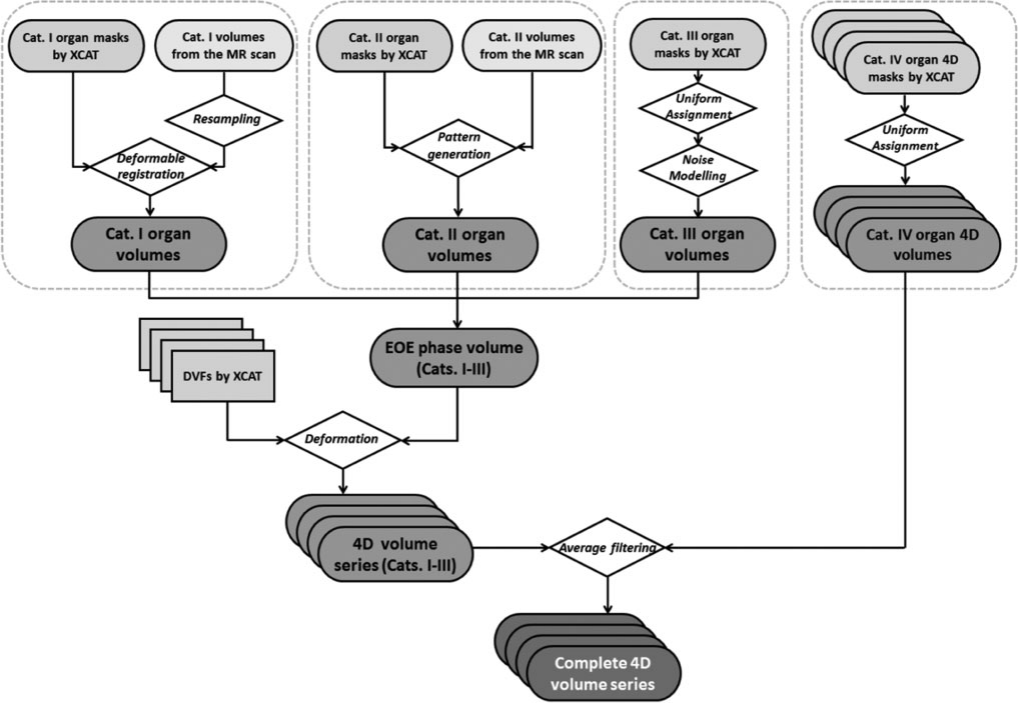
The general workflow of organ/soft tissue modeling of categories I to IV structures.

The overall workflow of the method is shown in Figure 2 as a guideline of Methods section. Generally, the structures in the defined imaging FOV were classified into 4 categories and were processed differently to generate images with synthesized MR texture at the reference phase, which was then used to generate 4-D MRI volumes afterward. Lists of the structures classified for each category are shown in Table 1. Details about the process of generating MRI images for each category of organs are explained subsequently.

#### Structures in category I

Category I structures were considered as key structures with unique anatomical details, and these structures were modeled that were defined in the clinical scan. Each structure was first assigned with a uniform base intensity value A. Next, the structure’s corresponding MR volume contoured in the clinical scan was resampled and registered to the structure’s binary mask from XCAT in Velocity AI software (Varian Medical Solutions, Palo Alto, California) using deformable multipass algorithm.^22^ The intensity values of the deformed MR volume underwent a linear transformation to set the volume’s average intensity as B (patient-specific average intensity value). The adjusted MR volume with the anatomical details was then superimposed to the structure’s uniform intensity in the reference volume. The resultant structure in the reference volume had an average intensity value of A + B. This “A + B” design allows customizable adjustment of the anatomical details’ weights on the final appearance of the phantom. The provided A and B values are listed in Table 1. The sum A + B values were measured from the selected patient, and the B values were the generalized results of measured organ intensity variations in multiple patients’ VIBE images in our radiation oncology clinic.

A spherical hypointensity tumor with a radius of *r* = 1.5 cm was added to the upper right liver portion (not shown in the Figure 2). To achieve a smooth liver/tumor transition, the tumor’s radial profile was modeled as a fourth-order polynomial, and the tumor’s center intensity value was set about 100 lower than the nearby liver voxels’ intensity values.

#### Structures in category II

Since category II structures listed in Table 1 have no particular anatomical landmarks inside their large volumes, a pattern generation method was adopted to build nonspecific soft tissue textures within the structures. For each structure of interest (SOI), the following steps describe the pattern generation process:

1. A volume mask of 5 × 5 × 5 voxels was randomly selected within the SOI.
2. A small ROI of the same size was randomly selected within the contoured muscle region in the clinical MR scan.
3. The small volume in the clinical MR scan was mapped to the volume mask in the SOI.
4. Steps 1 to 3 were iteratively implemented. The volume mask selection in step 1 was regulated by a constraint to make sure that each voxel would only be selected once during the iteration. The iteration stopped when no more 5 × 5 × 5 volume masks could be found within the SOI.
5. The volume mask size was reduced to 3 × 3 × 3. Steps 1 to 4 were repeated to generate patterns for voxels that were not included by 5 × 5 × 5 volume masks. The iteration stopped when no more 3 × 3 × 3 volume masks could be found within the SOI.
6. The volume mask size was reduced to 1 × 1 × 1. Steps 1 to 5 were repeated to generate patterns for voxels that were not included in previous iterations. The iteration stopped when all voxels in the SOI were modeled.
7. A 5 × 5 × 5 moving average filter was applied to the SOI’s simulated MR volume for improving the smoothness.

Finally, the simulated MR volumes of category II structures were integrated into the reference volume using the aforementioned A + B method with the specified values in Table 1.

#### Structures in category III

Category III structures in the reference volume were assigned with a uniform value A listed in Table 1. For the lungs and intestinal air, a simulated MR noise profile following Rayleigh distribution was added.^23^ The simulated mean noise level was empirically set as 5 for both structures.

#### Structures in category IV

Since the XCAT does not consider the mechanical properties of different tissue and potential interactions of different tissue types at their boundaries, the deformation vector fields (DVFs) describing deformations at structures’ surfaces are not fully continuous. This issue is more severe at the bone/soft tissue surfaces when using the DVF for motion modeling. To alleviate this issue, the structures in category IV were firstly modeled as “muscle” or “body” in the reference EOE volume, depending on the structures’ adjacencies to “muscle” or “body.” After the 4-D generation in the section “Generating a Synthetic 4-D MRI Volume Series,” these structures were assigned with uniform intensities at each phase volume (including EOE phase) separately.

### Generating a Synthetic 4-D MRI Volume Series

#### Four-dimensional volume series of categories I to III

As shown in Figure 2, 4-D volumes of the structures in categories I to III were generated by deforming the reference volume at EOE phase based on the backward DVFs from XCAT. These DVFs were generated by XCAT program at vector mode, and some key technical details for its implementation can be found in Supplementary Material I. The DVFs were visually checked and basic morphological repairs including filling isolated holes and removing spur voxels were implemented if necessary. The volumes at other phases were then generated using the processed DVFs and cubic interpolation method.

#### Four-dimensional volume series with category IV included

In each phase volume, the binary masks of category IV structures were identified. Each category IV structure was assigned with a uniform intensity value A listed in Table 1. The static spine marrow modeled at the reference phase was also inserted into each phase volume to avoid potential DVF errors at spine marrow boundaries. To fuse the spine/spine marrow surface for better texture continuity, an image average filtering using 3 × 3 × 3 moving average filter was included. Finally, a custom-designed weighted 5 × 5 × 5 moving average kernel (see Supplementary Material I) was applied to the whole volume except spine/spine marrow surface to improve soft tissue smoothness and MR visual reality.^24^

A total of three 4-D MR series were generated with large amplitude breath (maximum diaphragm motion = 3.0 cm, maximum chest anterior–posterior [AP] motion = 2.0 cm), normal breath (maximum diaphragm motion = 2.0 cm, maximum chest [AP] motion = 1.2 cm), and limited amplitude breath (maximum diaphragm motion = 2.0 cm, maximum chest AP motion = 0.5 cm). All image processing works were carried out in the MATLAB environment (R2014b; MathWorks Inc, Natick, Massachusetts) on a workstation with 16-GB RAM and a 3.4-GHz clock rate.

## Results

**Figure 3.**
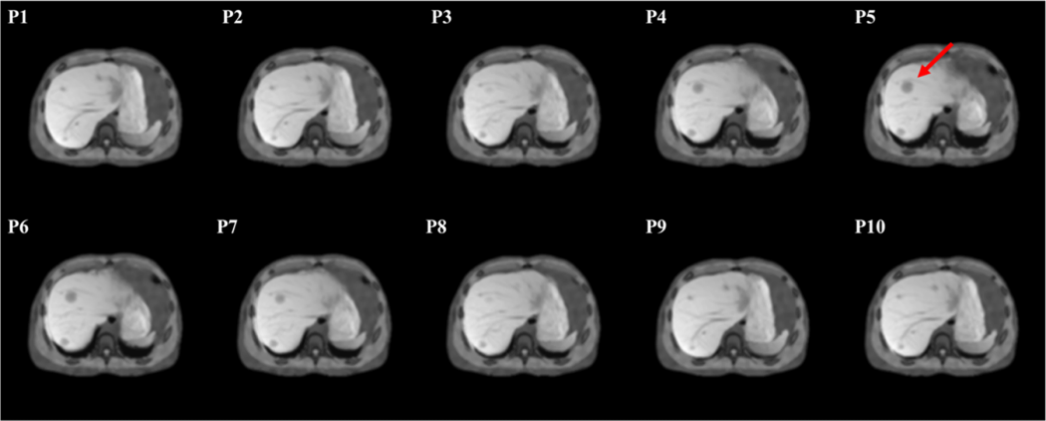
An example slice of the simulated magnetic resonance (MR) motion phantom at an axial view. The numbers at the upper left corner of each slice indicate the phase number during the motion. The red arrow in P5 indicates the spherical tumor’s position.

**Figure 4.**
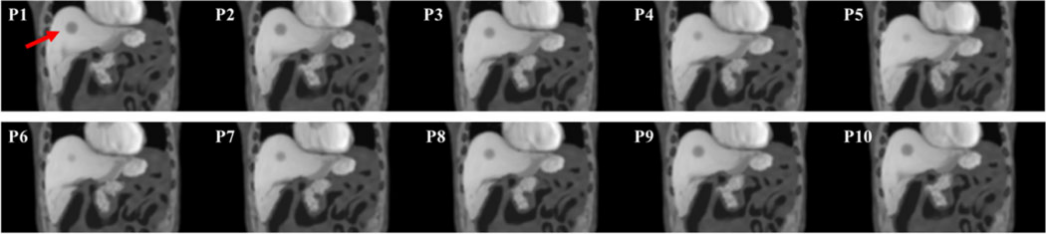
An example slice of the simulated magnetic resonance (MR) motion phantom at a coronal view. The numbers at the upper left corner of each slice indicate the phase number during the motion. The red arrow in P1 indicates the spherical tumor’s position.

Figures 3 and 4 show example slices of all 10 phases containing the spherical tumor at the axial view and coronal view, respectively. This 4-D series was simulated with a normal breath (maximum diaphragm motion = 2.0 cm, maximum chest AP = 1.2 cm), and the red arrows in the figures indicate the tumor’s position. The simulated images compare very favorably to actual clinical MR image appearance. Within the liver, the detailed anatomical landmarks and soft tissue patterns render a good illustration of the liver’s variation during the motion. In addition, different soft tissue regions are smoothly connected, and the transition between bony structures and soft tissue are well modeled without visual aliasing.

**Figure 5.**
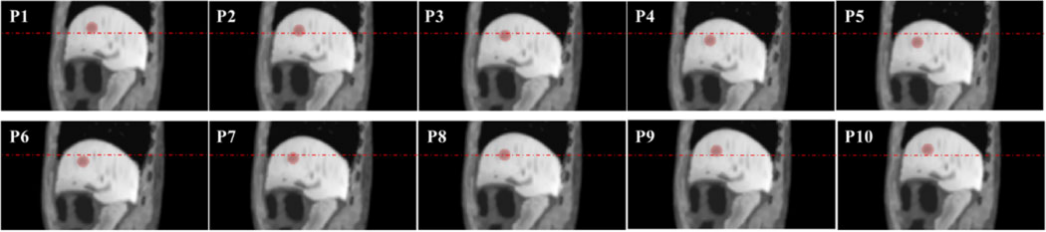
An example slice of the simulated magnetic resonance (MR) motion phantom at a sagittal view. The numbers at the upper left corner of each slice indicate the phase number during the motion. The red contours indicate the spherical tumor’s position. The horizontal dash lines provide a reference to appreciate the tumor’s superior–inferior motion.

Figure 5 shows example slices of all 10 phases at the sagittal view with tumor contours expressed by red shades. The tumor’s motion can be easily appreciated at the sagittal view with the red horizontal marker lines. The measured tumor volume at P1 using VelocityAI software was 13.90 cm^3^, and the measured tumor volume had limited change (±0.11 cm^3^) during the motion. The tumor’s center of mass shift between EOE (P1) and end of inhalation (EOI; P5) measured by VelocityAI software was 1.92 cm on superior–inferior (SI) direction and 1.10 cm on AP direction. These results demonstrate the great potential that this MR motion phantom has for liver MR motion studies as highlighted in the Discussion section.

The liver motions in Figures 3 to 5 are also presented as animations in a GIF format in the Supplementary Document II. The constructed three 4-D MR series in this work in raw format are accessible through the direct request to the authors.

## Discussion

The developed 4-D computerized MRI phantom in this work can provide a computerized modeling tool of liver motion simulation with a real patient MR texture. Compared to the previous effort of MR motion simulation work using the XCAT activity mode, this MR motion phantom contains abundant unique anatomical details and soft tissue texture patterns.^13,19^ As such, it can provide a powerful tool to investigate emerging techniques of liver motion studies. The creation of population-averaged MR phantom based on multiple patients’ clinical image could be essential for potential use of MR simulation studies, including functional map generation, target auto-segmentation, and motion assessment. Such work requires much more efforts from clinical aspects and will be considered as prioritized direction for our future work.

### Structure Modeling

The structures in the defined XCAT FOV were classified into 4 categories with different processing methods. This approach was motivated by our observations in liver MR imaging for radiotherapy. Category I structures were considered as key structures because these structures have more patient-specific anatomical details and are commonly contoured as important organs at risk in liver radiotherapy. To simulate unique anatomical details within structures, the structures’ MR volumes in the clinical patient scan were mapped into the phantom using the VelocityAI software. The deformation from the clinical images to binary XCAT masks was successful. At the reference EOE phase, the volume difference between the deformed 7 category I organs and their binary masks was about 0.9% ±1.0%, which could be seen as indications of accurate deformation. During the 4-D generation, the processed DVFs from XCAT were adopted without further use of deformable registration.

Category II structures have no particular anatomical landmarks inside their large volumes. As a result, the MR textures in category II structures were simulated via an iterative pattern generation method in the section “Structures in category II.” The category III structures are associated with lungs and intestines. Since the air in lung and intestines has low signal intensities in T1-weighted MR imaging, uniform intensity assignment with noise modeling would be sufficient to simulate acceptable MR appearance in these structures. Category IV structures were modeled via the method described in the sections “Structures in category IV” and “Four-dimensional volume series with category IV included” to avoid potential errors at bone/soft tissue boundaries, which are explained in more details in the section “Challenges in XCAT DVF”. In the XCAT phantom, the tumor’s inclusion is optional, and it has to be generated separately from the rest of the body structures. As such, the optional spherical tumor in this work was not categorized into any structure group. The tumor’s size and location could be adjusted to simulate different liver diseases that may acquire different imaging strategies for tumor motion tracking for radiotherapy purpose. In addition, the tumor’s motion can be edited separately as in XCAT to simulate desynchronized (in reference to the liver) tumor motion or irregular tumor trajectory.^25^

**Figure 6.**
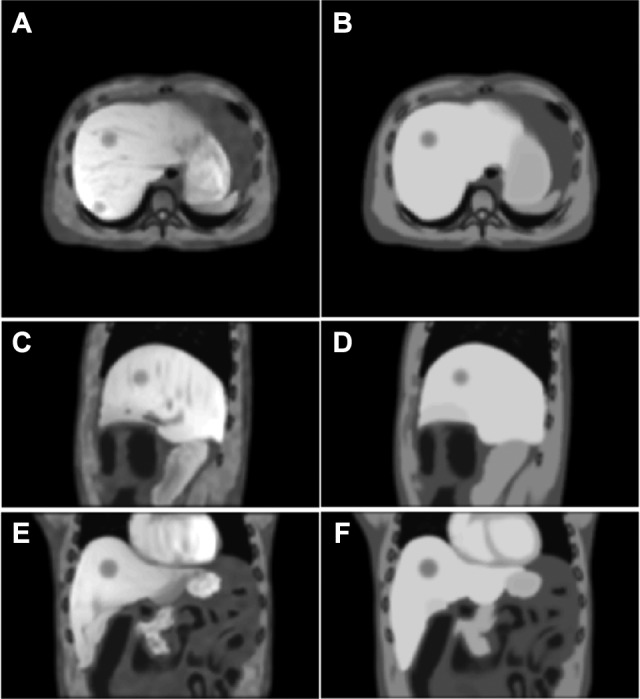
A comparison of the end of exhalation (EOE) volume between the developed magnetic resonance (MR) phantom (left column) and the pseudo-MR volume generated by the current extended cardiac-torso (XCAT) phantom with uniform intensity assignment (right column) at axial (A and B), sagittal (C and D), and coronal (E and F) views.

With the described structure modeling methods, the illustration of the developed phantom matches closely to the clinical MR images. Figure 6 shows a comparison of the EOE volume between the developed MR phantom and the pseudo-MR volume generated by the current XCAT phantom with uniform intensity assignment. The developed phantom (left column) is more realistic than the pseudo-MR volume in terms of overall image appearance and textured details in the organs and muscle.

### Phantom Flexibility

**Figure 7.**
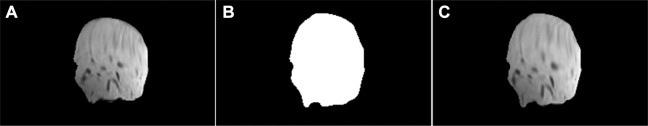
An example of deforming a reference structure magnetic resonance (MR) volume to an enlarged liver volume. A, A sagittal slice of the liver MR at a reference organ geometry. B, The binary mask of an enlarged liver volume. C, The generated deformed liver MR volume using the Demons method.

Based on the current version of the XCAT software, the MR motion phantom in this work has a great deal of flexibility. For example, the phantom’s motion pattern can be modeled with patient-specific motion patterns that are derived from external surrogates for radiation treatment.^26^ The users are also allowed to edit the forward DVF based on the reference volume to get other phase volumes.^27^ In addition, users can adjust the tumor’s position, shape, and motion patterns through the XCAT program. With additional knowledge of XCAT use, the users could build their customized version of the phantom with altered phantom geometry or specific organ motion trajectories based on the provided materials of our phantom. To construct a phantom with a new phantom geometry, a set of 4-D structure mask series can be generated with altered XCAT input, and the structures should be remodeled in the workflow as described in Figure 2. As a simple implementation with less effort of processing clinical data, the Demons algorithm can be adopted as a simple approach to transform the MR volumes of category I structures at the reference anatomical geometry into a new one.^28^ Such operation should be performed for each category I structure standalone. Figure 7 shows an example of generating enlarged liver MR volume (V = 2200.00 mL) from the reference liver MR volume (V = 1975.90 mL) using our in-house Demons platform. Specifically, the enlarged liver MR volume (C) can be generated by registering the reference liver MR volume (A) to the enlarged liver mask (B). The result image in (C) preserves key image features of (A) with enlarged area. Then categories II to IV structures that are free of unique anatomical details can be remodeled without clinical data processing. The MR reference volume at a new patient geometry can then be synthesized by integrating all remodeled structures. A set of muscle MR volume from an anonymized clinical scan is available at (Link will be provided later) for soft tissue pattern generation of category II structures.

### Challenges in XCAT DVF

In this phantom, the organs’ motion was implemented with DVFs exported from XCAT vector mode. Instead of registering the extracted clinical anatomy of category I organs to each respiratory phase, the utilization of XCAT DVF allows the accurate rendering of organ deformation without the introduction of uncertainties of deformable registrations during at each phase.^29^ In addition, the tumor motion can be edited separately with customized (not necessarily clinical relevant) trajectory via XCAT vector mode to simulate complicated motion scenario. Hence, the DVF fields can be seen as the ground truth for the designed motion scenario for validation purpose.

**Figure 8.**
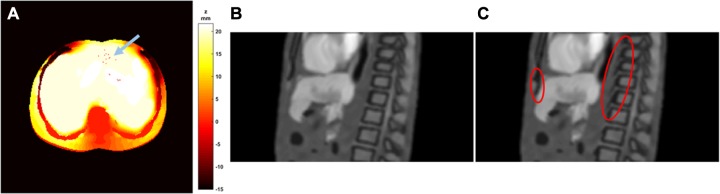
Examples of an imperfect deformation vector field (DVF). A, An axial slice of the Z-displacement (superior–inferior [SI]) in millimeters at the end of inhalation (EOI) phase. The blue arrow indicates some scattered “holes” in the smooth region. B, A sagittal slice of the EOI phase volume showing bone/muscle connectivity. C, A sagittal slice of aliasing EOI phase volume when bony structures in category IV are modeled in the same way as category III in the end of exhalation (EOE) phase volume only. Red circles indicate the aliased spine and cortical bone caused by the DVF discontinuity.

The DVF generated by XCAT, however, is not perfect. In the XCAT program, organs are modeled by NURBS surfaces with a limited number of control points. When generating a volume at an arbitrary time point in the 4-D series, the positions of each organ’s control points are generated with a low-resolution DVF, and the organs’ positions are determined by the derived control points.^3^ Since the organs are modeled as homogeneous compositions, this approach works well as the intraorgan variation during organ motion is not relevant in the XCAT. However, to describe organ motions in the MR appearance with many intraorgan anatomical details, a full-resolution DVF describing each voxel’s displacement is mandatory. In this work, the DVFs exported by the XCAT vector mode were generated via the interpolation of the control points’ DVF. Because of the aforementioned discontinuity in the DVF modeling and the potential calculation error, the interpolated full-resolution DVF may have minor defects. Figure 8A shows an example slice of the Z-displacement (SI direction) at the EOI phase. As can be seen, some scattered “holes” are found in the area within the uniform Z-displacement area. These minor errors may not cause noticeable aliasing in the deformed MR volumes, but proper corrections can be included for a more robust workflow. Such errors can be corrected with basic image morphological operations as stated in the Methods section.

As mentioned above, category IV structures and the static marrow in category II had to be modeled at each phase volume in a manner that is different from the rest of the structures. This approach was selected to deal with the DVF discontinuity problem at the bone/soft tissue surfaces. If category IV structures were modeled only in the reference (EOE) volume as category III, the deformations of spine and cortical bone in category IV at the other phase volumes suffer incorrect morphology. As an example, Figure 8C shows a sagittal view of the EOI phase volume when category IV structures were modeled in the same way as category III structures in the reference (EOE) volume. Because of the DVF discontinuity, some areas near the bone/soft tissue surfaces were void without proper information. This led to the dark spurs (“motion residues”) of the spine and the cortical bone (indicated by red circles). In contrast, when using the presented method for bony structure modeling in the Methods section, the bone/muscle surfaces in Figure 8B are well defined without noticeable aliasing. Although the DVF approach is not perfect, it is the most common approach for clinical deformable image registration and has been incorporated into clinical standard workflow. An advanced solution will be the finite element-based modeling with physiological basis for organ interaction, and we are seeing this solution as a valuable working direction in the future.

## Conclusion

In this work, a 4-D computerized MRI phantom was developed for liver motion studies with synthetic MR textures from real patient images. The developed phantom provides a valuable tool for liver 4-D MR imaging research development and evaluation.

## Supplementary Material

Supplementary material

Supplementary material

## Abbreviations

AP: anterior–posterior
CT: computed tomography
4-D: 4-dimensional
DVF: deformation vector field
EOE: end of exhalation
EOI: end of inhalation
FOV: field of view
MRI: magnetic resonance imaging
NURBS: nonuniform rational B-splines
ROI: region of interest
SI: superior–inferior
SOI: structure of interest
VIBE: volumetric interpolated breath-hold exam
XCAT: extended cardiac-torso.
