# Supplementary material for: Development of a Computerized 4-D MRI Phantom for Liver Motion Study
Source: Technol Cancer Res Treat. 2017 Aug 9;16(6):1051–9. doi: 10.1177/1533034617723753 (PMC5575982; doi:10.1177/1533034617723753)
Supplement: Supplementary material [file SupDoc1.docx]

**Supplementary Document I**

**Reference input file for XCAT program**

*mode = 0 # (0 = phantom, 1 = heart lesion, 2 = spherical lesion, 3 = plaque, 4 = vectors, 5 = save anatomical variation)*

*act_phan_each = 1*

*atten_phan_each = 0*

*act_phan_ave = 0*

*atten_phan_ave = 0*

*motion_option = 2 # (0=beating heart only, 1=respiratory motion only, 2=both motions)*

*out_period = 5*

*time_per_frame = 0*

*out_frames = 10*

*hrt_period = 1 # hrt_period (SECS)*

*hrt_start_ph_index = 0.0*

*heart_base = vmale50_heart.nrb*

*heart_curve_file = heart_curve.txt*

*apical_thin = 0.0*

*uniform_heart = 0*

*hrt_v1 = 0.0*

*hrt_v2 = 0.0*

*hrt_v3 = 0.0*

*hrt_v4 = 0.0*

*hrt_v5 = 0.0*

*hrt_t1 = 0.5*

*hrt_t2 = 0.192*

*hrt_t3 = 0.115*

*hrt_t4 = 0.193*

*resp_period = 5 # resp_period (SECS)*

*resp_start_ph_index = 0.0 # resp_start_phase_index (range=0 to 1, full exhale= 0.0, full inhale=0.4*

*max_diaphragm_motion = 2.0*

*max_AP_exp = 1.2*

*dia_filename = diaphragm_curve.dat*

*ap_filename = ap_curve.dat*

*hrt_motion_x = 0.0*

*hrt_motion_y = 1.2*

*hrt_motion_z = 2.0*

*hrt_motion_rot_xz = 0.0*

*hrt_motion_rot_yx = 0.0*

*hrt_motion_rot_zy = 0.0*

*vessel_flag = 0*

*coronary_art_flag = 0*

*coronary_vein_flag = 0*

*papillary_flag = 0*

*arms_flag = 0*

*gender = 0*

*organ_file = vmale50.nrb*

*phantom_long_axis_scale = 1.0*

*phantom_short_axis_scale = 1.0*

*phantom_height_scale = 1.0*

*head_cir_scale = 1.0*

*head_height_scale = 1.0*

*head_skin_cir_scale = 1.0*

*torso_long_axis_scale = 1.0*

*torso_short_axis_scale = 1.0*

*chest_skin_long_axis_scale = 1.0*

*chest_skin_short_axis_scale = 1.0*

*abdomen_skin_long_axis_scale = 1.0*

*abdomen_skin_short_axis_scale = 1.0*

*pelvis_skin_long_axis_scale = 1.0*

*pelvis_skin_short_axis_scale = 1.0*

*arms_cir_scale = 1.0*

*arms_length_scale = 1.0*

*arms_skin_cir_scale = 1.0*

*legs_cir_scale = 1.0*

*legs_length_scale = 1.0*

*legs_skin_cir_scale = 1.0*

*bones_scale = 1.0*

*head_torso_muscle_scale = 1.0*

*arms_muscle_cir_scale = 1.0*

*legs_muscle_cir_scale = 1.0*

*hrt_scale = 1.0*

*breast_type = 1*

*which_breast = 0*

*breast_long_axis_scale = 1.0*

*breast_short_axis_scale = 1.0*

*breast_height_scale = 1.0*

*br_theta = 10.0*

*br_phi = 0.0*

*r_br_tx = 0.0*

*r_br_ty = 0.0*

*r_br_tz = 0.0*

*l_br_tx = 0.0*

*l_br_ty = 0.0*

*l_br_tz = 0.0*

*rdiaph_liv_scale = 1.0*

*ldiaph_scale = 1.0*

*marrow_flag = 1*

*thickness_sternum = 0.4*

*thickness_scapula = 0.35*

*thickness_ribs = 0.3*

*thickness_backbone = 0.4*

*thickness_pelvis = 0.4*

*thickness_collar = 0.35*

*thickness_humerus = 0.45*

*thickness_radius = 0.45*

*thickness_ulna = 0.45*

*thickness_hand = 0.35*

*thickness_femur = 0.5*

*thickness_tibia = 0.6*

*thickness_fibula = 0.5*

*thickness_patella = 0.3*

*thickness_foot = 0.4*

*thickness_si = 0.6*

*thickness_li = 0.6*

*si_air_flag = 1*

*li_air_flag = 5*

*thickness_esoph = 0.3*

*# set the volume of the organs; (0 = do not change)*

*vol_prostate = 0.0*

*vol_testes = 0.0*

*vol_liver = 0.0*

*vol_stomach = 0.0*

*vol_pancreas = 0.0*

*vol_spleen = 0.0*

*vol_gall_bladder = 0.0*

*vol_rkidney = 0.0*

*vol_lkidney = 0.0*

*vol_radrenal = 0.0*

*vol_ladrenal = 0.0*

*vol_small_intest = 0.0*

*vol_large_intest = 0.0*

*vol_bladder = 0.0*

*vol_thyroid = 0.0*

*vol_thymus = 0.0*

*vol_salivary = 0.0*

*vol_pituitary = 0.0*

*vol_eyes = 0.0*

*vol_rovary = 0.0*

*vol_lovary = 0.0*

*vol_ftubes = 0.0*

*vol_uterus = 0.0*

*vol_vagina = 0.0*

*vol_larynx = 0.0*

*vol_trachea = 0.0*

*vol_esoph = 0.0*

*vol_epidy = 0.0*

*pixel_width = 0.16719 # pixel width (cm);*

*slice_width = 0.16719 # slice width (cm);*

*array_size = 256*

*subvoxel_index = 1*

*startslice = 666 # start_slice;*

*endslice = 815 # end_slice;*

*d_ZY_rotation = 0*

*d_XZ_rotation = 0*

*d_YX_rotation = 0*

*X_tr = 0.0*

*Y_tr = 0.0*

*Z_tr = 0.0*

*activity_unit = 0*

*# edit here to label each organ*

*myoLV_act = 2*

*myoRV_act = 2*

*myoLA_act = 2*

*myoRA_act = 2*

*bldplLV_act = 2*

*bldplRV_act = 2*

*bldplLA_act = 2*

*bldplRA_act = 2*

*coronary_art_activity = 2*

*coronary_vein_activity = 2*

*valve_thickness = 0.1*

*body_activity = 2*

*skin_activity = 2*

*rbreast_activity = 2*

*lbreast_activity = 2*

*muscle_activity = 2*

*brain_activity = 2*

*sinus_activity = 2*

*liver_activity = 2*

*gall_bladder_activity = 2*

*r_lung_activity = 2*

*l_lung_activity = 2*

*esophagus_activity = 2*

*esophagus_cont_activity = 2*

*laryngopharynx_activity = 2*

*larynx_activity = 2*

*st_wall_activity = 2*

*st_cnts_activity = 2*

*pancreas_activity = 2*

*r_kidney_cortex_activity = 2*

*r_kidney_medulla_activity = 2*

*l_kidney_cortex_activity = 2*

*l_kidney_medulla_activity = 2*

*adrenal_activity = 2*

*r_renal_pelvis_activity = 2*

*l_renal_pelvis_activity = 2*

*spleen_activity = 2*

*rib_activity = 2*

*cortical_bone_activity = 2*

*spine_activity = 2*

*spinal_cord_activity = 2*

*bone_marrow_activity = 2 ;*

*art_activity = 2*

*vein_activity = 2*

*bladder_activity = 2*

*prostate_activity = 2*

*asc_li_activity = 2*

*trans_li_activity = 2*

*desc_li_activity = 2*

*sm_intest_activity = 2*

*rectum_activity = 2*

*sem_activity = 2*

*vas_def_activity = 2*

*test_activity = 2*

*penis_activity = 2*

*epididymus_activity = 2*

*ejac_duct_activity = 2*

*pericardium_activity = 2*

*cartilage_activity = 2*

*intest_air_activity = 2*

*ureter_activity = 2*

*urethra_activity = 2*

*lymph_activity = 2*

*lymph_abnormal_activity = 2*

*trach_bronch_activity = 2*

*airway_activity = 2*

*uterus_activity = 2*

*vagina_activity = 2*

*right_ovary_activity = 2*

*left_ovary_activity = 2*

*fallopian_tubes_activity = 2*

*parathyroid_activity = 2*

*thyroid_activity = 2*

*thymus_activity = 2*

*salivary_activity = 2*

*pituitary_activity = 2*

*eye_activity = 2*

*lens_activity = 2*

*lesn_activity = 2*

*Corpus_Callosum_act = 2*

*Caudate_act = 2*

*Internal_capsule_act = 2*

*Putamen_act = 2*

*Globus_pallidus_act = 2*

*Thalamus_act = 2*

*Fornix_act = 2*

*Anterior_commissure_act = 2*

*Amygdala_act = 2*

*Hippocampus_act = 2*

*Lateral_ventricle_act = 2*

*Third_ventricle_act = 2*

*Fourth_ventricle_act = 2*

*Cerebral_aqueduct_act = 2*

*Mamillary_bodies_act = 2*

*Cerebral_peduncles_act = 2*

*Superior_colliculus_act = 2*

*Inferior_colliculus_act = 2*

*Pineal_gland_act = 2*

*Periacquaductal_grey_outer_act = 2*

*Periacquaductal_grey_act = 2*

*Pons_act = 2*

*Superior_cerebellar_peduncle_act = 2*

*Middle_cerebellar_peduncle_act = 2*

*Substantia_nigra_act = 2*

*Medulla_act = 2*

*Medullary_pyramids_act = 2*

*Inferior_olive_act = 2*

*Tegmentum_of_midbrain_act = 2*

*Midbrain_act = 2*

*cerebellum_act = 2*

*white_matter_act = 2*

*grey_matter_act = 2*

*energy = 120 # radionuclide energy in keV*

*#--------------------Regional Heart Motion Defect---------------------------*

*motion_defect_flag = 0*

*#---------------------Heart lesion parameters------------------------------*

*ThetaCenter = 0.0*

*ThetaWidth = 100.0*

*XCenterIndex = .5*

*XWidthIndex = 60*

*Wall_fract = 1.0*

*motion_scale = 0.0*

*border_zone_long = 10*

*border_zone_radial = 5*

*#---------------------Spherical lesion parameters--------------------------*

*x_location = 87 # x coordinate (pixels) to place lesion*

*y_location = 113 # y coordinate (pixels) to place lesion*

*z_location = 105 # z coordinate (pixels) to place lesion*

*lesn_diameter = 30.0 # Diameter of lesion (mm)*

*#---------------------Heart plaque parameters------------------------------*

*p_center_v = 0.2*

*p_center_u = 0.5*

*p_height = 1.0*

*p_width = 2.0*

*p_length = 5.0*

*p_id = aorta*

*#---------------------Vector parameters------------------------------------*

*vec_factor = 2 # higher number will increase the precision of the vector output*

**Backward deformation vector field (DVF) generation**

In this work, the phase volumes were generated by deforming the reference volume (EOE phase) with the backward DVF that aligned phase volumes to EOE volume. The respiratory cycle was set to 5 seconds, and the time interval between two adjacent phase volumes is 0.5 second. Let 0.0 represents EOE phase index and 0.1, 0.2,....0.9 represent other phases’ indices. To generate the DVF of a certain phase volume between 0.1 and 0.9, these edits in the XCAT input file are necessary:

1. *Change ‘Program Mode’ to 4 (Vector mode)*
2. *Set ‘time_per_frame’ to 5 – phase index * 5*
3. *Set ‘resp_start_ph_index’ to phase index*
4. *Set ‘vec_factor’ to 2. Although higher number will increase the precision of the vector output, a number larger than 2 may lead to extremely long calculation*

**Weighted moving average filtering kernel**

*
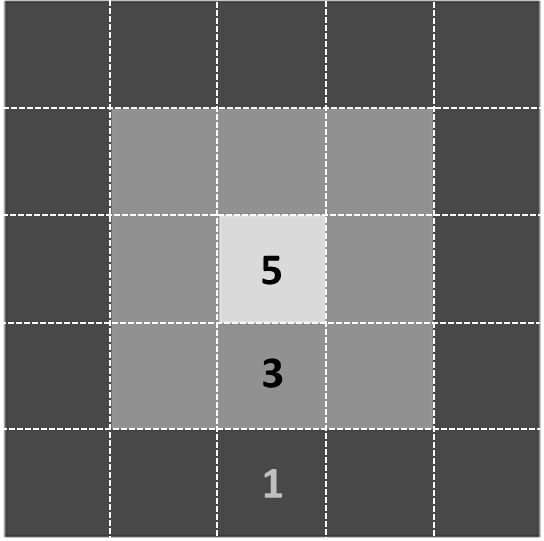
*

Figure S1: The weighted moving average filtering kernel for improving soft tissue smoothness. The kernel is built in a 3D fashion with 5x5x5 dimension
