# Supplementary figures and images for: Development of a Computerized 4-D MRI Phantom for Liver Motion Study
Source: Technol Cancer Res Treat. 2017 Aug 9;16(6):1051–9. doi: 10.1177/1533034617723753 (PMC5575982; doi:10.1177/1533034617723753)

## Slide 1
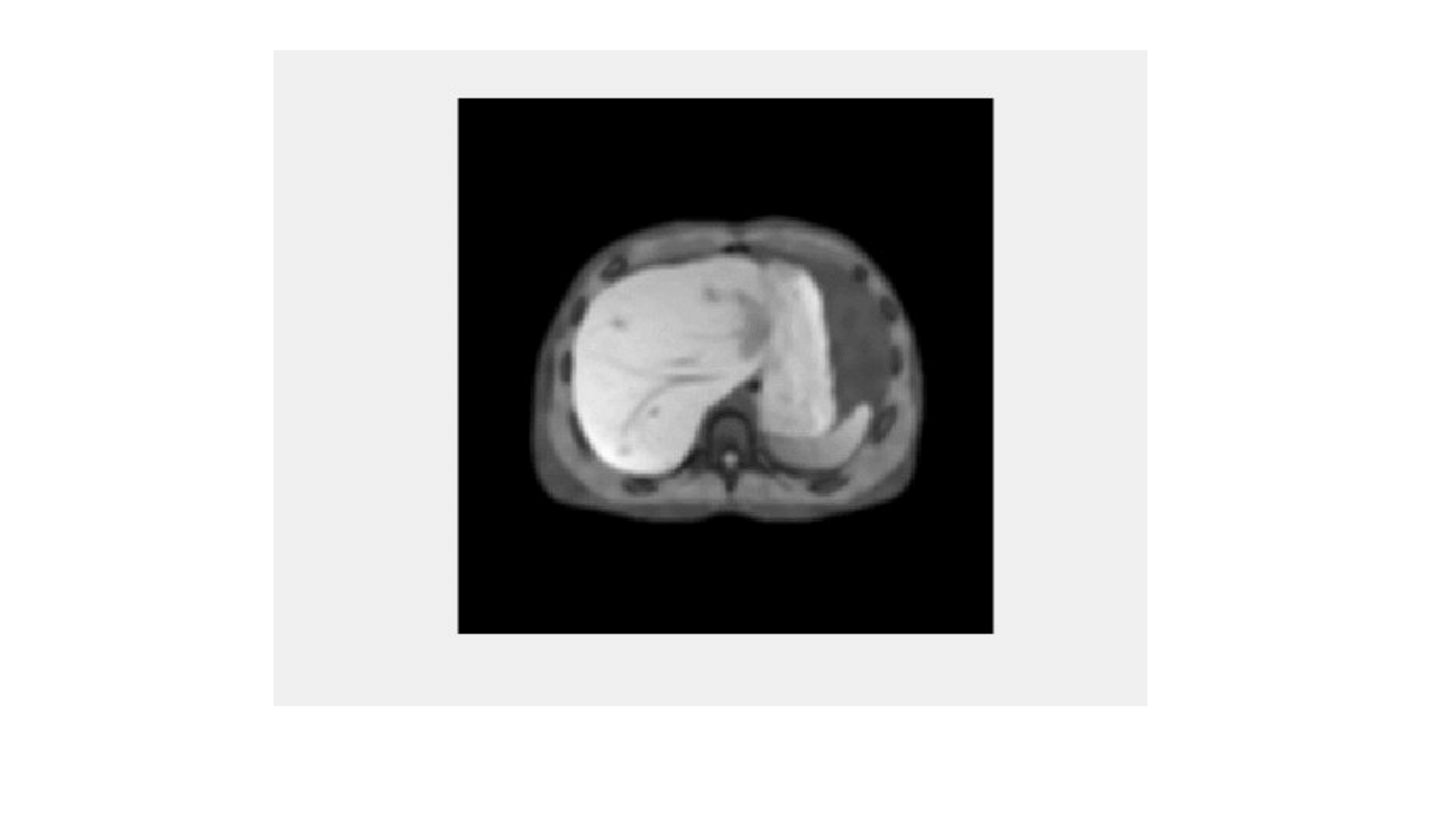

## Slide 2
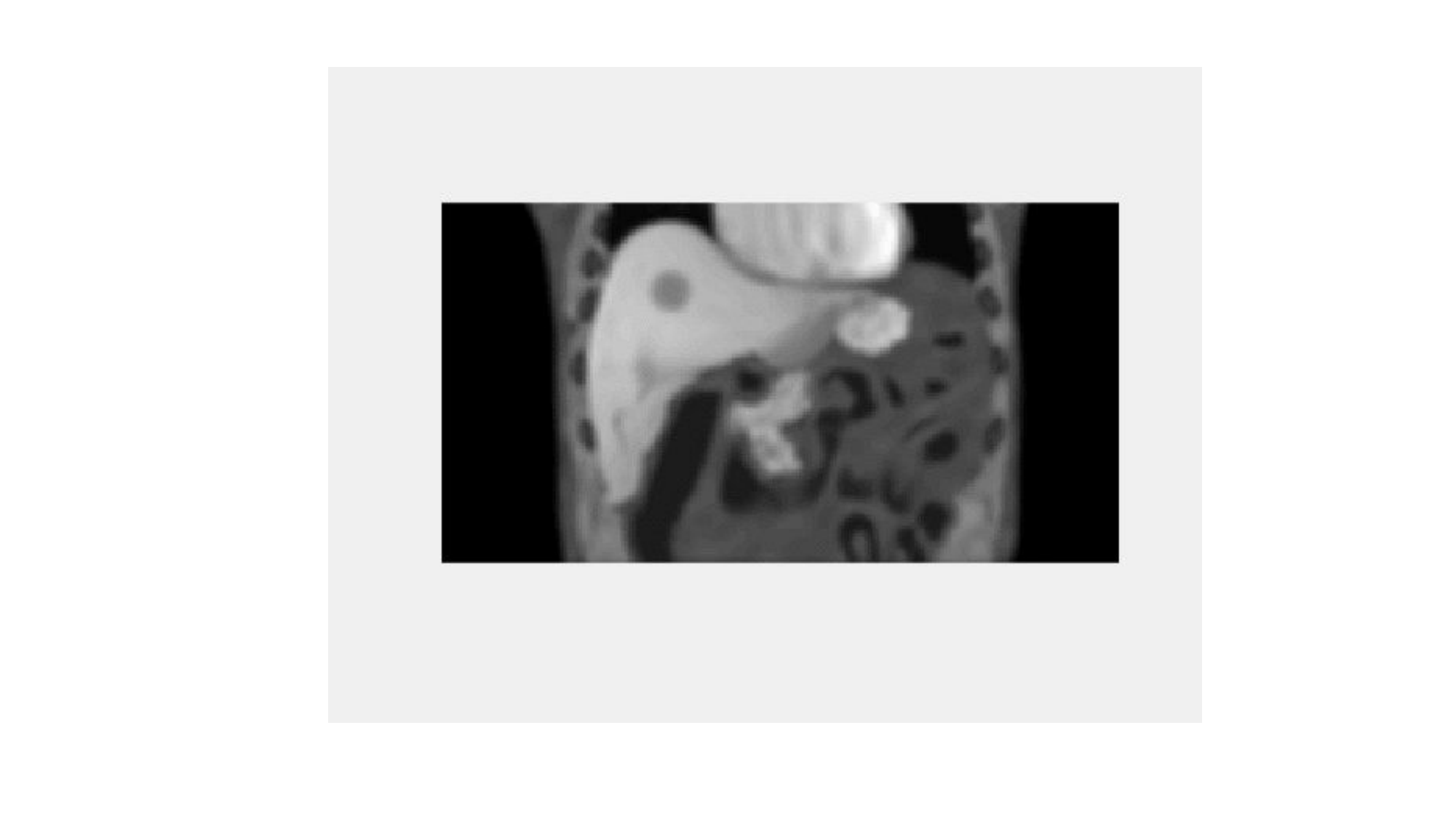

## Slide 3
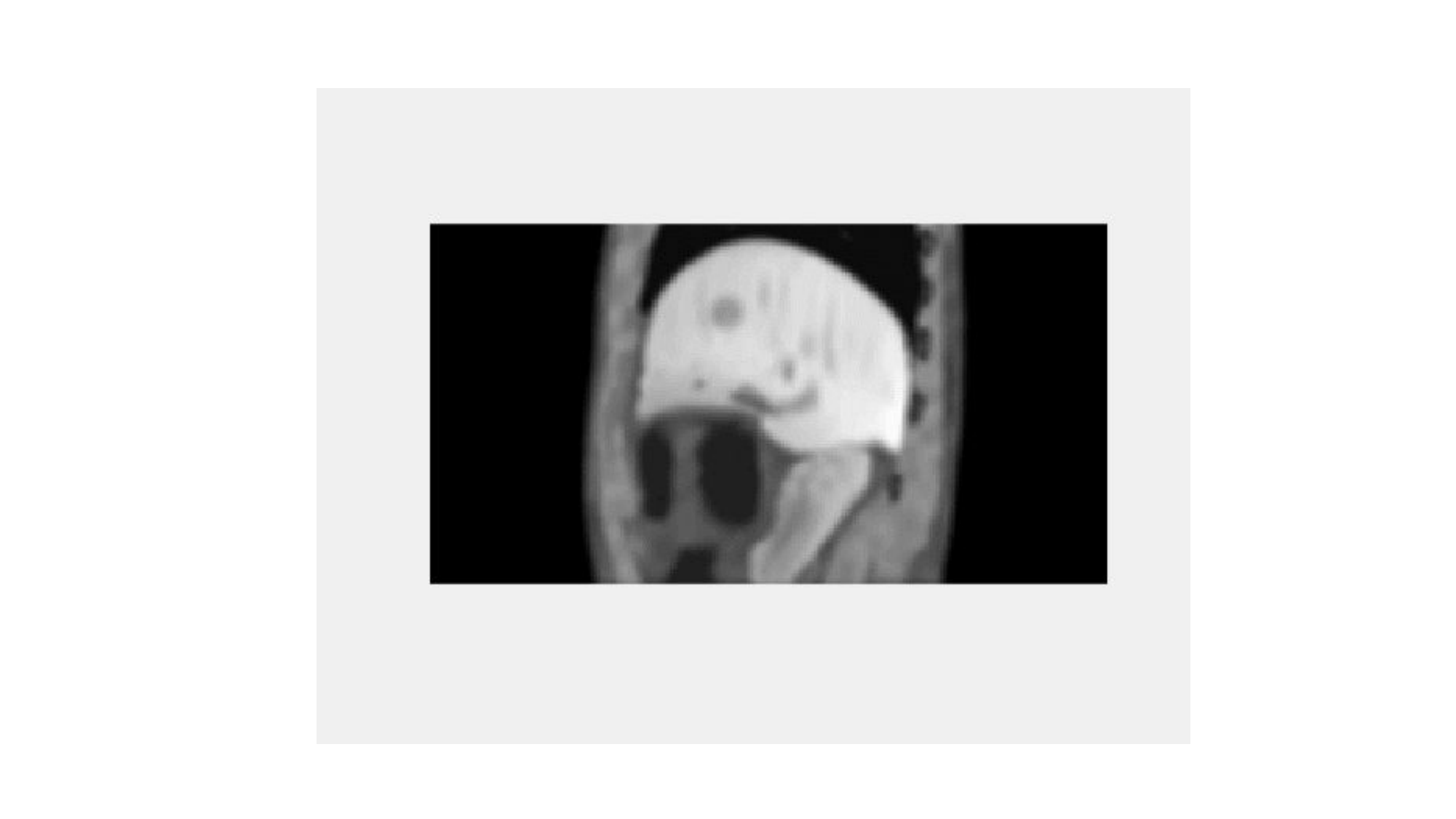

Supplement: Supplementary material [file SupII.pptx]
